# Gender- and Age-Related Differences of Ocular Biometric Parameters in Patients Undergoing Cataract Surgery in Bosnia and Herzegovina

**DOI:** 10.1155/2023/1950257

**Published:** 2023-11-21

**Authors:** Edita Zvorničanin, Zoran Vatavuk, Maja Popović, Jasmin Zvorničanin

**Affiliations:** ^1^Private Healthcare Institution Vase Zdravlje, Tuzla 75000, Bosnia and Herzegovina; ^2^Department of Ophthalmology, University Clinical Centre “Sestre Milosrdnice”, Eye Clinic, Zagreb 10000, Croatia; ^3^Cancer Epidemiology Unit, Department of Medical Sciences, University of Turin, CPO-Piemonte, Turin 10125, Italy; ^4^Department of Ophthalmology, University Clinical Centre Tuzla, Tuzla 75000, Bosnia and Herzegovina; ^5^Faculty of Health Studies, University of Bihać, Bihać 77000, Bosnia and Herzegovina

## Abstract

**Purpose:**

The aim of the study is to determine the distribution and mutual relationship of ocular biometric parameters, as well as to evaluate gender- and age-related differences in patients undergoing cataract surgery in Bosnia and Herzegovina.

**Materials and Methods:**

It was a retrospective cross-sectional study of consecutive patients who underwent cataract surgery between January 2017 and December 2021 in a tertiary care clinic. All biometric measurements were performed using the optical biometer OA-2000 (Tomey, Nagoya, Japan).

**Results:**

The study evaluated 1278 eyes from 1278 consecutive cataract patients. The average age of all included patients was 69.4 ± 9.98 (range 40–96). A total of 672 eyes (52.58%) were from females. The mean axial length (AL), anterior chamber depth (ACD), lens thickness (LT), and mean keratometry were 23.46 ± 1.18 mm, 3.17 ± 0.40 mm, 4.54 ± 0.48 mm, and 43.42 ± 1.55D, respectively. Corneal astigmatism of ≥1D, >2D and >3D was found in 33.4%, 7.8% and 2.5% patients, respectively. Females were found to have shorter AL (*p* < 0.0001), shallower ACD (*p* < 0.0001) and steeper corneas (*p* < 0.0001). In both genders, AL, ACD and with the rule astigmatism showed a decreasing trend (*p* = 0.0001), while keratometry, the average cylinder, and against the rule astigmatism showed an increasing trend (*p* = 0.0001) with increasing age. Furthermore, in both genders, there was an increasing trend in ACD (*p* = 0.0001), and a decreasing trend in keratometry (*p* = 0.0001) and LT (*p* = 0.0001) with increasing AL.

**Conclusions:**

This study provides useful reference data on ocular biometry for cataract surgeons in Bosnia and Herzegovina. Female patients tend to have steeper corneas, shorter AL and shallower AC than males, and these differences are independent of age or AL.

## 1. Introduction

Cataract represents the leading cause of preventable blindness in the world [1]. The current standard for the management of a visually significant cataract is the surgical removal of the cataractous lens and its replacement with an intraocular lens (IOL) [2]. To meet increasing patient demands and expectations for postoperative results, cataract surgery is constantly evolving with improved surgical techniques, IOL designs, and calculation methods [3]. Accurate measurement of ocular biometric parameters is essential for predictable postoperative refractive results [4], and novel optical biometric devices have been introduced with a higher level of precision than ultrasonic biometry [5]. Therefore, with adequate preoperative IOL calculation, cataract surgery also has the ability to correct refractive errors [6].

Several previous studies have reported the biometric characteristics of cataract surgery candidates from different parts of the world. Age-related changes in corneal astigmatism prevalence and characteristics, as well as ocular biometric parameters, have been documented [7–22]. Furthermore, there have been certain differences in ocular biometry between male and female patients reported [7, 8, 11, 13–22]. However, studies that specifically address gender differences in different age groups are scarce [17]. To the best of our knowledge, there are no data available on the characteristics of ocular biometric parameters in Bosnia and Herzegovina, and there is a lack of studies from Balkan and eastern Europe in general describing similar study populations.

The purpose of this study was to determine the distribution and mutual relationship of ocular biometric parameters, as well as to evaluate the gender- and age-related differences in patients undergoing cataract surgery in Bosnia and Herzegovina. To interpret and discuss our findings, we performed an extensive literature review of similar studies conducted in different contexts and populations. The findings of this study will provide useful data for both health professionals and industry to appropriately allocate resources and meet future demands.

## 2. Materials and Methods

This was a retrospective cross-sectional study of the eyes of 1278 consecutive patients who underwent cataract surgery in a private tertiary care clinic in Bosnia and Herzegovina, between January 1, 2017 and December 31, 2021. All included patients were citizens of Bosnia and Herzegovina and belong to different ethnic groups. However, there is no major genetic difference between three major ethnic groups in Bosnia and Herzegovina, (Bosniaks, Bosnian Serbs, and Bosnian Croats), indicating that they present the same gene pool [23]. Furthermore, the human population of Bosnia and Herzegovina is closely related to other populations in the Balkans [23]. In patients who underwent surgery on both eyes, we used data for the right eye only. The current study was approved by the institution's Ethics Committee (Approval number: 01-1-2022). Informed consent was obtained from all patients included in the study at the time of cataract surgery, and the study was conducted according to the Declarations of Helsinki.

All biometric measurements were performed using the optical biometer OA-2000 (Tomey, Nagoya, Japan). For measurements of axial length (AL), anterior chamber depth (ACD) and lens thickness (LT), it uses swept-source optical coherence tomography (SS-OCT) with a laser wavelength of 1060 nm [24]. It is capable of simultaneously measuring corneal curvature by Placido-disc–based topography techniques with 9 rings each 256 points in a 5.5 mm zone projected onto the cornea [25]. For measurement, all subjects were asked to sit in front of the instrument, place their chin in the chinrest, focus on a target, blink completely and keep eyes wide open without blinking during scanning. The device performs the measurements automatically without the need for realignment.

Corneal astigmatism was designated as against-the-rule (ATR) when the axis of correcting minus cylinder was within 30° of the vertical 90° meridian, with-the-rule (WTR) when the correcting minus cylinder axis was within 30° of the horizontal 180° meridian, and oblique (OBQ) if it was neither ATR nor WTR orientation. Based on AL all eyes were stratified into 3 groups: shorter than 22.0 mm, 22–24.5 mm, and longer than 24.5 mm [12]. Age was stratified into 5 groups: 40–49, 50–59, 60–69, 70–79, and ≥80 years of age [8, 19].

After biometry, all patients underwent a complete ophthalmic examination, including visual acuity testing with Snellen charts, tonometry, detailed slit lamp biomicroscopy, pupillary reaction, and fundoscopy. The inclusion criteria for the current study were the presence of cataract and the age of 40 years or older. The exclusion criteria were dense cataracts that interfered with biometric measurement, ocular surface disorders, previous corneal or intraocular surgery, and a history of intraocular inflammation and penetrating eye trauma.

Continuous variables were tested for normality using the Shapiro–Wilk test and presented as mean and standard deviation (SD) or, if strongly skewed, additionally as medians and interquartile range (IQR). Differences between continuous variables among different groups were tested using the *t*-test if normally distributed, while the non-parametric Wilcoxon Mann–Whitney test was used for non-normally distributed variables. Binary and categorical variables are reported as absolute numbers and percentages and are tested for differences using the chi-square test. A rank-based non-parametric Kruskal–Wallis test was applied to determine differences between more than two groups of an independent variable in continuous dependent variables. The correlation was estimated using the pairwise Pearson correlation coefficient. All analyses were performed with Stata version 15.1 (StataCorp, College Station, Texas, USA). The significance level for hypothesis testing was set at 0.05.

## 3. Results

This study evaluated 1278 eyes from 1278 consecutive cataract patients. The average age of all included patients was 69.4 ± 9.98 (range 30–96) years. The average age of the male and female patients was 68.6 ± 10.0 and 70.1 ± 9.9 years, respectively (*p* = 0.009). A total of 672 eyes (52.58%) were from female patients and 820 right eyes (64.16%) were included in the analysis. Corneal astigmatism of ≥1D, >2D and >3D was found in 438 (34.3%), 108 (8.5%) and 34 (2.7%) patients, respectively. Female patients had shorter AL than male patients in all age groups (*p* < 0.0001) (Tables 1 and 2). Furthermore, female patients had a shallower ACD than males in four of five age groups (*p* < 0.0001). The flat and steep keratometry readings were steeper in female patients in four of the five age groups (*p* < 0.0001). On the other hand, the average power of the astigmatism cylinder was insignificantly lower in female patients (*p* = 0.284). The prevalence of astigmatism greater than 1D, 2D, or 3D was similar in both genders (all *p* values >0.22). However, male patients had a higher percentage of ATR astigmatism compared to females (*p* < 0.0001).

**Figure 1:**
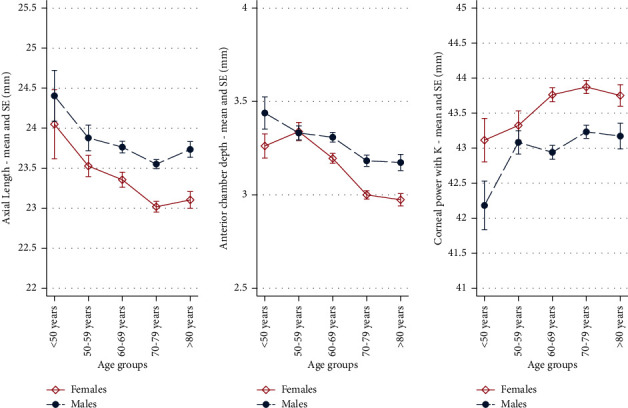
Age- and gender-related differences in ocular biometric parameters. SE-standard error.

**Figure 2:**
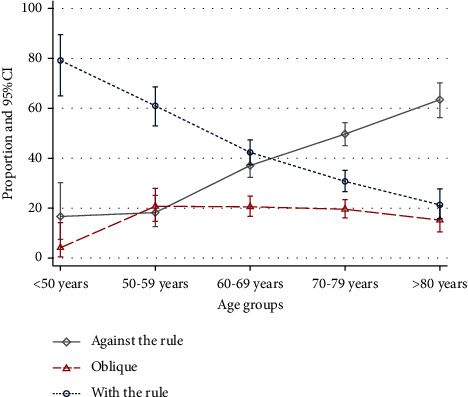
Corneal astigmatism by age groups. CI-confidence interval.

AL and ACD showed a decreasing trend with increasing age (*p* = 0.0001), and these findings were consistent for both males and females (Figure 1). On the other hand, the flat and steep keratometry showed an increasing trend with increasing age (*p* = 0.0001 and *p* = 0.0005). Furthermore, the average cylinder power also gradually increased with age (*p* = 0.0001). Corneal astigmatism continuously changed its orientation with age, where WTR astigmatism presented decreasing and ATR astigmatism increasing trend with increasing age (*p* = 0.0001) (Figure 2). All the aforementioned age-related changes were similar in both genders. There were no significant differences in astigmatism orientation in relation to AL (*p* = 0.48) and ACD (*p* = 0.108). The cylinder power was the largest among eyes with ATR astigmatism (median 0.84, range 0.05–9.94), and slightly lower among eyes with WTR astigmatism (median 0.73, range 0.05–8.46), with *p* value = 0.004. The lowest cylinder power was found in eyes with OBQ astigmatism (median 0.6, range 0–8.12), with statistically significant differences from the other two types (both *p* values <0.0001).

In both genders, there was an increasing trend in ACD with increasing AL (*p* = 0.0001) (Tables 3 and 4). On the other hand, there was a decreasing trend of both keratometry values (*p* = 0.0001) and LT (*p* = 0.0001) with increasing AL. Cylinder power and frequency were increased in patients with AL shorter than 22.0 mm or longer than 24.5 mm (*p* = 0.003). There was an overall difference in the prevalence of astigmatism ≥1D between eyes with AL <22.0 mm (44.6%), eyes 22.0–24.5 mm (31.8%) and eyes >24.5 mm (40.6%) (*p* = 0.006). Furthermore, we observed similar differences between the axial length and the prevalence of astigmatism >2D (*p* = 0.001) and >3D (*p* = 0.003).

## 4. Discussion

This study evaluated the distribution and mutual relationship of the ocular biometric parameters and the characteristics of corneal astigmatism in candidates for cataract surgery from Bosnia and Herzegovina. So far, there are only two studies published from this region analyzing corneal astigmatism in patients undergoing cataract surgery [6, 26]. To the best of our knowledge, this is the first study from Bosnia and Herzegovina that focused on preoperative assessment of both corneal astigmatism and biometric parameters in cataract surgery patients. The results of this study are obtained from a homogenous group of consecutive patients over a relatively long period of time and should represent normative data for cataract surgery patients in Bosnia and Herzegovina, as well as in neighboring regions.

Optical biometry provides a significantly higher percentage of cases within ±0.50D of postoperative refractive error compared to ultrasound biometry [27]. Despite the introduction of new technologies, partial coherence interferometry (PCI) is still considered a gold standard for measuring ocular biometric parameters [25, 28]. The OA-2000 uses SS-OCT technology for ocular biometry and has provided excellent repeatability and reproducibility in both healthy and cataractous eyes [24, 25, 28]. These favorable results have been validated in direct comparison with standard PCI and other biometers [25]. However, it should be borne in mind that regardless of the accuracy of these devices, both PCI and SS-OCT biometers are not able to measure ocular biometric parameters through dense cataracts where ultrasound biometry still has a significant value [15, 25].

Like other organs of the human body, the eyes exhibit differences between females and males [17]. As previously reported, female patients in this study demonstrated shorter AL with consequently shallower ACD and steeper corneas than male patients [7, 8, 11, 13–21]. Furthermore, this is the first study that has documented all aforementioned gender-related differences according to different age groups. These results have important clinical implications, as women tend to report a worse subjective visual function than men, before and after surgery [29]. This clinical finding could be associated with shorter AL and a steeper cornea found in female patients, which could amplify minor differences in ocular measurements or intraocular lens calculation, and therefore result in lower precision in biometry prediction [29]. Male patients were also presented with a higher percentage of ATR astigmatism, which is consistent with the results of previous studies finding that ATR astigmatism increased more with age in males than in females [17, 30]. The rationale for these differences could be found in the fact that male cataract surgery candidates have greater height and weight, which can explain certain biometric differences [8, 11, 13–16, 19, 20]. Another explanation may be the consequence of the differences in genetics and sex hormones, where aging can exert different effects in the two genders [14, 17, 19]. This is in accordance with the observation that gender could be considered as an independent predictor of postoperative refraction error in different IOL formulas [31].

In this study, we have found that the AL exhibits a decreasing trend with age, which is consistent with the result of previously published studies [8, 12, 13, 15, 17, 19–21]. At the same time, we observed an increasing trend in keratometry values [8, 12, 13, 17–21]. There is quite a simple explanation for this trend, i.e., an emmetropic state could be derived from a steep cornea coupled with a relatively short AL, or a flat cornea combined with a relatively long AL [17, 20]. Thus, while AL is highly associated with genetic and environmental factors, there is a relatively coordinated eye growth and active emmetropization process [32]. Therefore, it is hypothesized that new generations grow in height and consequently have longer AL and steeper corneas than the old ones [8, 13, 17]. Another possible explanation is that younger cataract patients are more likely to have myopia [8, 13, 19].

The results of recent studies presented significant racial differences. Studies from Asian and Hispanic populations have presented longer axial length than European populations [33]. All patients in our study were of European origin, [23] and our biometric results are the closest to those from Germany [7] and Slovakia [20]. However, the interpretation of potential ocular biometric differences is complex and would require adjustment for the refraction, height, age, and even the level of education of the study population [11]. However, most of the recent studies reported a greater ACD in males, younger patients, and patients with greater AL [7, 8, 13, 15, 16, 19–21]. On the other side, we have also found a decreasing trend in ACD with steeper corneal curvature [7, 8, 11, 12]. Regardless of race, there is also a decreasing trend in ACD with age, which could be attributed to age-related lens thickening [8, 12, 13, 16, 18–22]. This highlights another supposed mechanism of emmetropisation, the lens thins (or decreases in power) as the eye gets longer (myopic) and thickens (or increases in power) as the eye gets shorter (hyperopic) [19]. Thus, most ocular biometric parameters have a pronounced mutual dependence, which is present in both genders and different races, but also reflects changes occurring during the lifetime.

The results of this study confirm that in both genders, the keratometry value, average cylinder power, astigmatism prevalence, as well as the ATR astigmatism prevalence increased with age [7, 8, 10, 12]. Astigmatism was more prevalent not only in patients with long eyes and steep corneas or short eyes with flat corneas [7] but also in younger and older patients [10, 12]. Some physiologic factors have been proposed to account for the age-related changes in the corneal curvature and these include a reduction in pressure of the eyelids, extraocular muscle tension, visual feedback, degenerative changes in the corneal tissue structure in elderly persons, and the effect of the intraocular pressure on the curvature of the cornea [8]. Furthermore, this is the first study which found significantly pronounced cylinder power in patients with ATR astigmatism, which additionally confirms the aforementioned age-related changes in the corneal curvature.

In an era of increasing patient expectations, cataract surgery provides a unique opportunity to correct corneal astigmatism at the time of surgery [6, 10, 12]. Based on these results, more aggressive ATR astigmatism treatment with the aim of its full correction should be considered, as its magnitude is likely to increase with age, especially in male patients [6, 10, 17]. However, the full correction of WTR astigmatism in younger patients' at the time of the cataract surgery could be misleading, given its probable progression to ATR astigmatism in years to come. Furthermore, two thirds of the patients in this study were presented with corneal astigmatism of less than 1D, where smaller incisions and incision positioning on the steep corneal axis could provide excellent refractive results [6, 12, 14]. With this individual approach, it would be possible to adjust cataract surgery and calculate the most suitable IOL power for each patient, which minimizes postoperative spectacle dependence [17].

However, this study has some limitations, including retrospective and clinically based design, which can lead to selection bias. Nevertheless, this study is based on a population of consecutive patients over a relatively long period, which ensures some generalizability to the underlying general population with similar clinical indications. In this study, we included only one eye from each patient, however; the same methodology was used in most previous studies [9, 10, 13, 15–20, 22]. We were unable to determine the relationship between refraction and ocular biometric parameters due to the cloudy crystalline lens of the cataracts. Another limitation of the current study is the lack of information on anthropometric characteristics and education level, factors that could also influence some of these parameters and their mutual correlations.

In conclusion, we report, for the first time, the distribution of ocular biometric parameters and corneal astigmatism, as well as their mutual correlations, in the population of cataract surgery candidates in Bosnia and Herzegovina. Female patients have steeper corneas, lower average cylinder power, higher prevalence of ATR astigmatism, shorter AL, and shallower AC than males. These gender-related differences were present regardless of age or AL. This study also supports the general trend of age-related decrease in AL and ACD, and the increase in the magnitude and prevalence of astigmatism in cataract patients. In an era of increasing patient expectations, it is necessary to anticipate gender- and age-related differences to achieve the best possible postoperative results.

## Figures and Tables

**Table 1:** Ocular biometric parameters by gender.

Ocular biometric parameters	Total mean (SD) or *N* (%)	Male mean (SD) or *N* (%)	Female mean (SD) or *N* (%)	*p* value^1^
Keratometry (D)				
K1	42.93 (1.64)	42.57 (1.54)	43.25 (1.67)	**<0.0001**
K2	43.90 (1.57)	43.55 (1.54)	44.22 (1.53)	**<0.0001**
K	43.42 (1.55)	43.07 (1.48)	43.74 (1.54)	**<0.0001**
Astigmatism (D)	0.97 (0.89)	0.98 (0.90)	0.97 (0.88)	0.874
ATR	546 (42.72)	288 (47.52)	258 (38.39)	**0.001**
OBQ	240 (18.78)	114 (18.81)	126 (18.75)	
WTR	492 (38.50)	204 (33.66)	288 (42.86)	
AL (mm)	23.46 (1.18)	23.73 (1.07)	23.22 (1.23)	**<0.0001**
ACD (mm)	3.17 (0.40)	3.25 (0.40)	3.10 (0.39)	**<0.0001**
LT (mm)	4.54 (0.48)	4.53 (0.49)	4.55 (0.47)	0.333

^1^Wilcoxon Mann–Whitney test. ACD-anterior chamber depth; AL-axial length; ATR-against the rule astigmatism; K1-flat keratometry; K2-steep keratometry; K-average keratometry; LT-lens thickness; OBQ-oblique astigmatism; SD-standard deviation; WTR-with the rule astigmatism. In bold are the statistically significant results.

**Table 2:** Ocular biometric parameters by gender and age group.

Study population by gender and age	Ocular biometric parameters mean (SD)						Astigmatism (D) median (IQR)
	#Eyes	AL (mm)	ACD	Corneal power (D)			
				K1	K2	K	
40–49 years							
Male	24	24.40 (1.55)	3.44 (0.42)	41.70 (1.64)	42.66 (1.85)	42.18 (1.70)	0.64 (0.50–1.15)
Female	24	24.05 (2.11)	3.26 (0.32)	42.34 (1.49)	43.88 (1.78)	43.11 (1.52)	1.28 (0.57–2.17)
Total	48	24.23 (1.84)	3.35 (0.38)	42.02 (1.59)	43.27 (1.90)	42.65 (1.67)	0.92 (0.50–1.79)
*p* value (sex)^1^		0.103	**0.046**	0.208	**0.016**	0.083	0.103
50–59 years							
Male	90	23.88 (1.53)	3.33 (0.35)	42.62 (1.62)	43.54 (1.63)	43.08 (1.58)	0.80 (0.41–1.38)
Female	69	23.53 (1.09)	3.34 (0.40)	42.87 (1.83)	43.78 (1.72)	43.32 (1.73)	0.77 (0.43–1.12)
Total	159	23.73 (1.36)	3.33 (0.38)	42.72 (1.71)	43.65 (1.66)	43.19 (1.65)	0.78 (0.42–1.25)
*p* value (sex)^1^		0.058	0.899	0.07	0.131	0.079	0.761
60–69 years							
Male	197	23.76 (1.02)	3.31 (0.36)	42.52 (1.40)	43.36 (1.47)	42.94 (1.39)	0.65 (0.41–1.01)
Female	202	23.36 (1.32)	3.20 (0.37)	43.30 (1.53)	44.23 (1.45)	43.76 (1.42)	0.72 (0.46–1.05)
Total	399	23.56 (1.20)	3.25 (0.37)	42.91 (1.52)	43.80 (1.52)	43.36 (1.46)	0.68 (0.43–1.02)
*p* value (sex)^1^		**<0.0001**	**0.003**	**<0.0001**	**<0.0001**	**<0.0001**	0.163
70–79 years							
Male	212	23.55 (0.81)	3.18 (0.44)	42.73 (1.43)	43.73 (1.43)	43.23 (1.36)	0.78 (0.48–1.32)
Female	263	23.02 (1.07)	3.00 (0.36)	43.41 (1.68)	44.33 (1.47)	43.87 (1.52)	0.72 (0.46–1.14)
Total	475	23.26 (1.00)	3.08 (0.41)	43.11 (1.61)	44.06 (1.48)	43.59 (1.48)	0.75 (0.46–1.20)
*p* value (sex)^1^		**<0.0001**	**<0.0001**	**<0.0001**	**0.0001**	**<0.0001**	0.163
≥ 80 years							
Male	83	23.73 (0.89)	3.17 (0.40)	42.51 (1.93)	43.83 (1.67)	43.17 (1.67)	1.01 (0.74–1.56)
Female	114	23.10 (1.11)	2.97 (0.36)	43.21 (1.73)	44.29 (1.62)	43.75 (1.63)	0.85 (0.54–1.34)
Total	197	23.37 (1.07)	3.06 (0.39)	42.91 (1.84)	44.10 (1.65)	43.51 (1.67)	0.93 (0.58–1.44)
*p* value (sex)^1^		**<0.0001**	**0.0003**	**0.029**	0.082	**0.048**	0.089
*p* value (age)^2^		**0.0001**	**0.0001**	**0.0002**	**0.002**	**0.001**	**0.0001**

^1^Wilcoxon Mann–Whitney test; ^2^difference between age groups, independent of gender-sex; Kruskal–Wallis test. ACD-anterior chamber depth; AL-axial length; K1-flat keratometry; K2-steep keratometry; K-average keratometry; SD–standard deviation; IQR–interquartile range. In bold are the statistically significant results.

**Table 3:** Ocular biometry by axial length.

Axial length (mm)	Ocular biometric parameters mean (SD)						Astigmatism (D) median (IQR)
	Eyes *N* (%)	ACD (mm)	Corneal power (D)			LT (mm)	
			K1	K2	K		
<22.0	69 (5.4)	2.79 (0.36)	45.28 (1.73)	46.50 (1.54)	45.89 (1.54)	4.72 (0.45)	0.85 (0.57–1.43)
22.0–24.5	1074 (84.0)	3.16 (0.39)	42.96 (1.45)	43.89 (1.35)	43.43 (1.34)	4.54 (0.49)	0.75 (0.45–1.14)
≥24.5	135 (10.6)	3.45 (0.30)	41.50 (1.58)	42.69 (1.71)	42.10 (1.53)	4.41 (0.38)	0.82 (0.51–1.49)
*p* value^1^	—	**0.0001**	**0.0001**	**0.0001**	**0.0001**	**0.0001**	**0.008**

^1^Kruskal–Wallis test. ACD-Anterior chamber depth; K1-flat keratometry; K2-steep keratometry; K-average keratometry; IQR-interquartile range; LT-lens thickness; SD-standard deviation. In bold are the statistically significant results.

**Table 4:** Correlation between ocular biometric parameters, age, and gender.

	Age	Gender	K1	K2	K	Cylinder	AL	ACD
Age	1.00	—	—	—	—	—	—	—
Gender	0.0727 **p** **=** **0.009**	1.00	—	—	—	—	—	—
K1	0.0823 **p** **=** **0.003**	0.2046 **p** **<** **0.0001**	1.00	—	—	—	—	—
K2	0.1131 **p** **<** **0.0001**	0.212 **p** **<** **0.0001**	0.8486 **p** **<** **0.0001**	1.00	—	—	—	—
K	0.1012 **p** **=** **0.0003**	0.2165 **p** **<** **0.0001**	0.9632 **p** **<** **0.0001**	0.9596 **p** **<** **0.0001**	1.00	—	—	—
Cylinder	0.0478 *p* = 0.088	−0.004 *p* = 0.897	−0.3501 **p** **<** **0.0001**	0.1985 **p** **<** **0.0001**	−0.0853 **p** **=** **0.0023**	1.00	—	—
AL	−0.1668 **p** **<** **0.0001**	−0.2129 **p** **<** **0.0001**	−0.4825 **p** **<** **0.0001**	−0.4771 **p** **<** **0.0001**	−0.4991 **p** **<** **0.0001**	0.0492 *p* = 0.079	1.00	—
ACD	−0.2516 **p** **<** **0.0001**	−0.1936 **p** **<** **0.0001**	−0.0721 **p** **=** **0.0099**	−0.0782 **p** **=** **0.0051**	−0.0781 **p** **=** **0.0052**	−0.0049 *p* = 0.860	0.3717 **p** **<** **0.0001**	1.00

ACD-anterior chamber depth; AL–axial length; K1-flat keratometry; K2-steep keratometry; K -average keratometry. In bold are the statistically significant results.

## Data Availability

The data set for this study has been uploaded to Dryad and is available via https://doi.org/10.5061/dryad.sbcc2fr7r.

## Additional Points

*Summary Statement*. Accurate measurement of ocular biometric parameters is essential for predictable postoperative refractive results, where gender- and age-related changes can play a significant role. Female patients have steeper corneas, lower average cylinder power, higher prevalence of against the rule astigmatism, shorter axial length, and shallower anterior chamber than males. There is also a general trend for an age-related decrease in axial length and anterior chamber and an increase in the magnitude and prevalence of astigmatism in cataract patients in Bosnia and Herzegovina.
